# Effectiveness of the Kano-AHP-TRIZ-TOPSIS integrated model for analyzing the healthcare emergency facility needs of the older adults

**DOI:** 10.3389/fpubh.2026.1817658

**Published:** 2026-04-28

**Authors:** Jiayi Yang, Jie Zhang, Chenlu Wang, Liping Liu, Kexin Wei, Yanling He

**Affiliations:** 1 Industrial Design Engineering Project, School of Art Design and Media, East China University of Science and Technology, Xuhui Campus, Shanghai, China; 2 School of Design and Art, Shanghai Institute of Mechanical and Electrical Engineering, Shanghai, China; 3 School of Design, Jiangnan University, Wuxi, China

**Keywords:** community environment, older adults design, older adults, public health, public service facilities, user requirements

## Abstract

**Background:**

This study focuses on the inadequate population aging adaptation of community emergency facilities under the backdrop of global population aging, which makes it difficult to support treatment of older adults within the “golden time” for sudden emergencies such as cardiogenic sudden death, stroke, and falls.

**Study design:**

A cross-sectional study was conducted from June 2024 to April 2025 in three typical old communities in Xuhui District, Shanghai: Huali Yuan, Meilong Village 1, and Meilong Village 2. Using 351 structured questionnaires and semi-structured interviews, the functional needs and usage barriers of older adults and caregivers regarding community emergency facilities were systematically collected. A Kano-AHP-TRIZ-TOPSIS integrated method framework was constructed to classify 19 needs by medical attributes, rank them by clinical priority, and resolve design contradictions.

**Results:**

An population aging community health emergency station prototype was ultimately developed. The study results showed that a “minimal emergency call mechanism” and a “clear visual and voice guidance system” were the core basic needs for older adults, with the “minimal emergency call” having the highest overall weight (0.410). Based on TRIZ theory, key conflicts related to functional integration, operational simplicity, and barrier-free space were effectively resolved. TOPSIS evaluation indicated that the overall performance of this prototype was significantly superior to that of traditional facility samples (*C_i_* = 0.894 compared to the sample *C_i_* = 0.167).

**Conclusion:**

This study demonstrates that the Kano-AHP-TRIZ-TOPSIS integrated model can be used to systematically convert complex user needs into design elements with priorities, and provides methodological support for the construction of prototypes of emergency stations in population aging communities. The proposed prototype achieved high overall preference under the evaluation context set in this study, showing design potential in emergency response, ease of use, and medical coordination. Moreover, the relevant results are mainly based on small-scale expert and user evaluations, reflecting the relative preference of the prototype scheme rather than direct evidence of actual application effects at the community level.

## 1 Introduction

The rise of the “Healthy Community” concept is a key indicator of the strategic shift in the construction of modern urban public health systems from “treatment-centered” to “health-centered” (1). Its core goal is to proactively intervene in the determinants of residents’ health through the creation of a multi-level, supportive physical and social environment, thereby comprehensively improving population health literacy and systematically managing the increasingly severe burden of chronic diseases and acute health risks (2, 3). This concept profoundly reflects the evolution of the medical model from the traditional biomedical model to a ‘bio-psycho-social-environmental’ integrated model. The World Health Organization, in the “Healthy Cities Initiative,” has repeatedly emphasized that effective health promotion must go beyond the traditional boundaries of passively providing medical services. Instead, it should rely on scientific built environment planning, stimulate broad community participation, and promote integrated health service innovations such as the “integration of medical treatment and prevention,” ultimately laying a solid community foundation for achieving universal health coverage (4–7).

From China’s practice, promoting the construction of healthy communities is an inevitable path to implement the “Healthy China 2030” strategic plan and optimize the allocation of public health resources (8). It requires integrating health into all policies, with particular attention to grassroots communities as the smallest management units, achieving the decentralization of medical resources and advancing health services (9). By embedding professional health support facilities (such as the first-aid stations studied in the article) into residents’ living circles, a continuous health management network of “family-community-hospital” three-tier linkage can be built, which is crucial for addressing the challenges of an aging population and reducing total social medical costs (10).

Globally, population aging has become an important issue facing public health and social governance, with the East Asian region being particularly typical. Compared with other regions, East Asian countries generally face a situation where low fertility rates (11), increased life expectancy, and weakened family caregiving functions coexist, leading to a faster aging process and more concentrated impacts. Japan, as one of the most aged countries in the world, had 29.3% of its population aged 65 and above by 2024; Republic of Korea is also rapidly entering a super-aged society, with the proportion of those aged 65 and above expected to reach 20.6% by 2025; China is similarly experiencing a deepening aging trend, with the population aged 60 and above reaching 280.04 million by the end of 2022, accounting for 19.8% of the total population. As population aging progresses, the older adults have gradually become a high-risk group for acute cardiovascular and cerebrovascular events, falls, and sudden respiratory events. These acute conditions are usually characterized by sudden onset, rapid progression, and short treatment windows. Taking acute myocardial infarction as an example, the “golden hour” at the early stage of onset directly determines the success rate of rescue and the quality of life prognosis (12–14); immediate assistance after a fall can also effectively prevent secondary injuries and complications. This places higher demands on community-level emergency response capabilities and health support systems.

Among them, the community, as the primary daily living and activity space for the older adults, has the allocation efficiency of its internal emergency resources directly related to the life safety of older adults residents in the event of acute incidents (15). Currently, there is a significant clinical adaptation gap in the construction of emergency facilities in most communities in China, meaning that there is an excessive focus on the physical deployment of equipment while severely neglecting specialized designs that match the physiological decline and cognitive characteristics of the older adults (16). Older adults individuals often have concurrent vision and hearing loss as well as declines in fine motor skills, yet existing emergency equipment generally has problems such as complex operation interfaces, unclear visual signs, and cumbersome procedural steps (17), making it difficult to be effectively used in critical situations such as sudden cardiovascular and cerebrovascular events (18). Furthermore, common public facilities equipped with devices like automated external defibrillators (AEDs) often have relatively single-function designs and fail to deeply integrate with clinical scenarios frequently seen in the older adults, such as arrhythmias, hypertensive crises, and hypoglycemic coma At the same time, these devices have isolated information and are unable to form effective data linkage with community residents’ electronic health records or family doctor service systems (19–20), thus failing to provide coherent medical support during critical emergency responses, greatly reducing their practical value in real pre-hospital emergency situations (21).

Therefore, under the framework of building healthy communities, it has become a key measure to establish a community emergency station centered on the clinical needs of older adults users that integrates life support and health monitoring and can achieve rapid intelligent response (22, 23). This is not only a core aspect of enhancing community health service capacity, but also an important practical path to promoting the national strategy of “healthy aging (25).” By implementing ergonomic design, integrating intelligent sensing technologies, and innovating service integration models, comprehensively improving the accessibility, usability, and clinical reliability of community emergency stations will provide a solid and effective solution for systematically responding to the public health challenges posed by an aging population (24).

**Figure 1:**
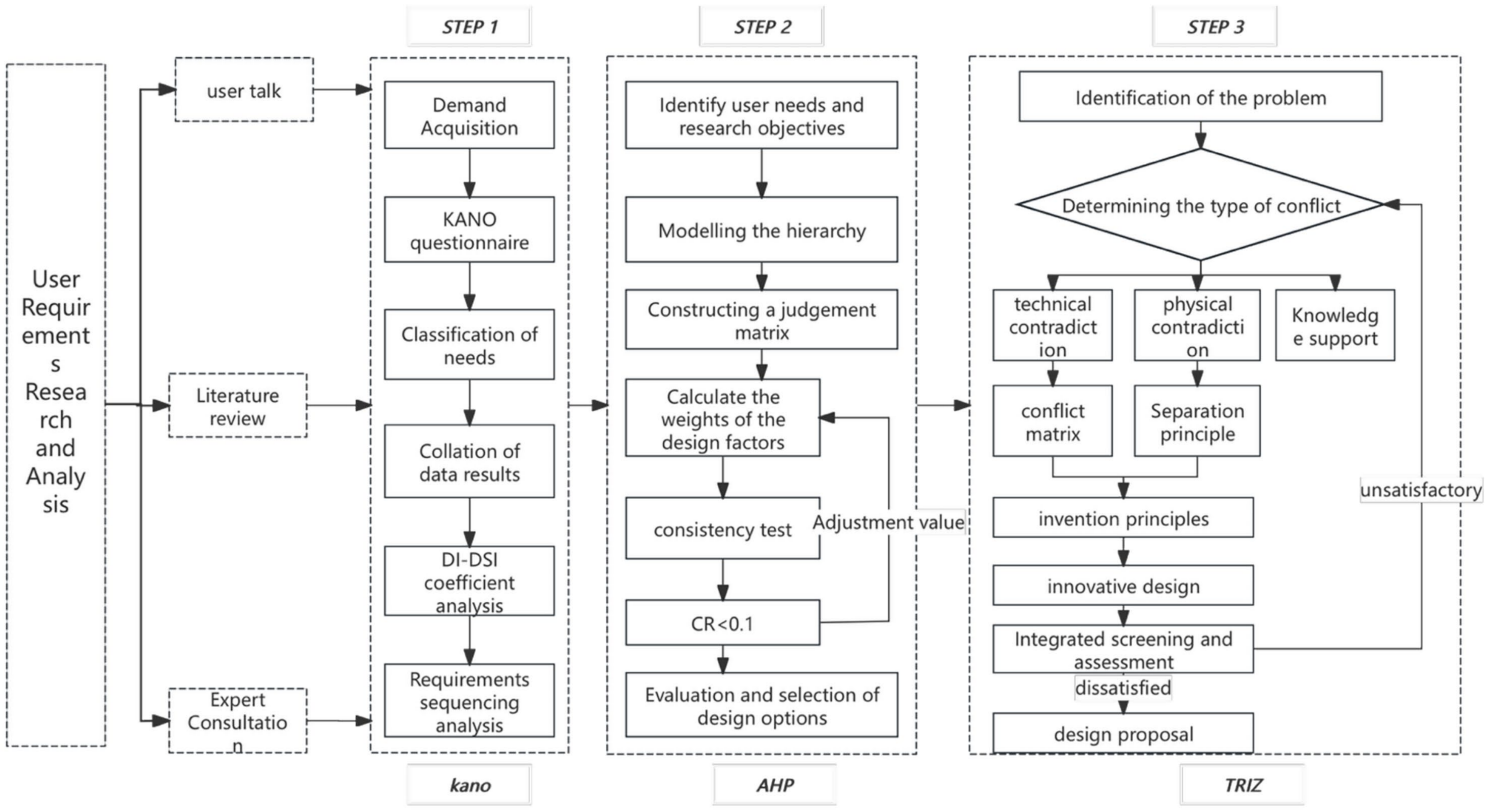
Kano-AHP-TRIZ technology roadmap.

Therefore, reliable data analysis and theoretical support should be provided (25, 28). In the process of establishing emergency stations in age-friendly communities, the Kano model (26–28), Analytic Hierarchy Process (AHP) (29–31), and TRIZ (32–35) theory are often used in combination (36–38). These three methods have significant advantages in different stages of design, as shown in Figure 1. The advantages of the Kano model in user demand analysis include: (i) establishing a clear hierarchical structure by categorizing user needs into basic, performance, and attractive types, making design solutions more precise; (ii) identifying the impact of different needs on user satisfaction through questionnaire analysis, optimizing product function configuration and improving user experience. The advantages of the AHP method in demand weight calculation include: (i) systematically decomposing complex requirements and building a multi-level structure model, making demand analysis more logical and intuitive; (ii) combining qualitative and quantitative analysis to calculate demand weights and priorities, enhancing the scientific basis of decision-making and avoiding subjective bias. The advantages of TRIZ theory in innovative design include: (i) effectively converting user needs into feasible technical solutions through tools such as the contradiction matrix and inventive principles, enhancing product innovation capability; (ii) resolving potential functional conflicts in the design process, optimizing product performance, and improving the feasibility of engineering implementation.

In summary, this study takes Huali Yuan Community, Meilong First Village, and Meilong Second Village in Xuhui District, Shanghai, as empirical research subjects. It integrates health and aging-friendly theories to innovatively construct a community health emergency station design under the Kano—AHP—TRIZ model and uses the Likert scale for the sustainable evaluation stage of the product (39). This study aims to address the following two research questions:

(i) What is the strategic design planning for the older adults in a healthy community?
(ii) What are the key elements of an aging-friendly health emergency station design based on the integrated model?

## 2 Research area and users

### 2.1 Study area

Huali Garden Community, Meilong First Village, and Meilong Second Village in Xuhui District, Shanghai, as typical old urban residential areas, are important practical areas for urban community renewal and age-friendly renovation. These communities were built relatively early (40), have limited public spaces, and a high proportion of older adults residents, forming a community population structure dominated by senior citizens. As the degree of aging continues to deepen, residents’ needs for daily health monitoring, emergency response, and convenient rest services are increasingly prominent. Although some basic public service facilities have been set up within the communities, there are still common problems such as scattered layout, single-function design, lack of intelligent support, and insufficient integration of local culture, which restrict the effective provision and user experience of community health services.

Based on this, this study takes Huali Yuan Community, Meilong First Village, and Meilong Second Village as empirical cases, focusing on the diversified health and safety needs of older adults residents, and explores optimization paths for community first aid stations in aspects such as intelligent response, environmental adaptability, and community integration (41). The aim is to build health service nodes with sustainable operational capabilities, intelligent service functions, and humanistic care characteristics, providing theoretical reference and practical experience for the updating of community health facilities in highly aging cities.

### 2.2 Research users

In order to fully understand the actual needs of the older adults for community health services, the research team conducted systematic field research in Hualiyuan Community, Meilong No. 1 Village and Meilong No. 2 Village in Xuhui District, Shanghai from June 2024 to April 2025. The research object is the older adults residents aged 60 and above in the community, their family members who live with them or caregivers who have been involved in their daily care for a long time, aiming at identifying the demand for community health services from the dual perspectives of direct users and potential supporters.

**Figure 2:**
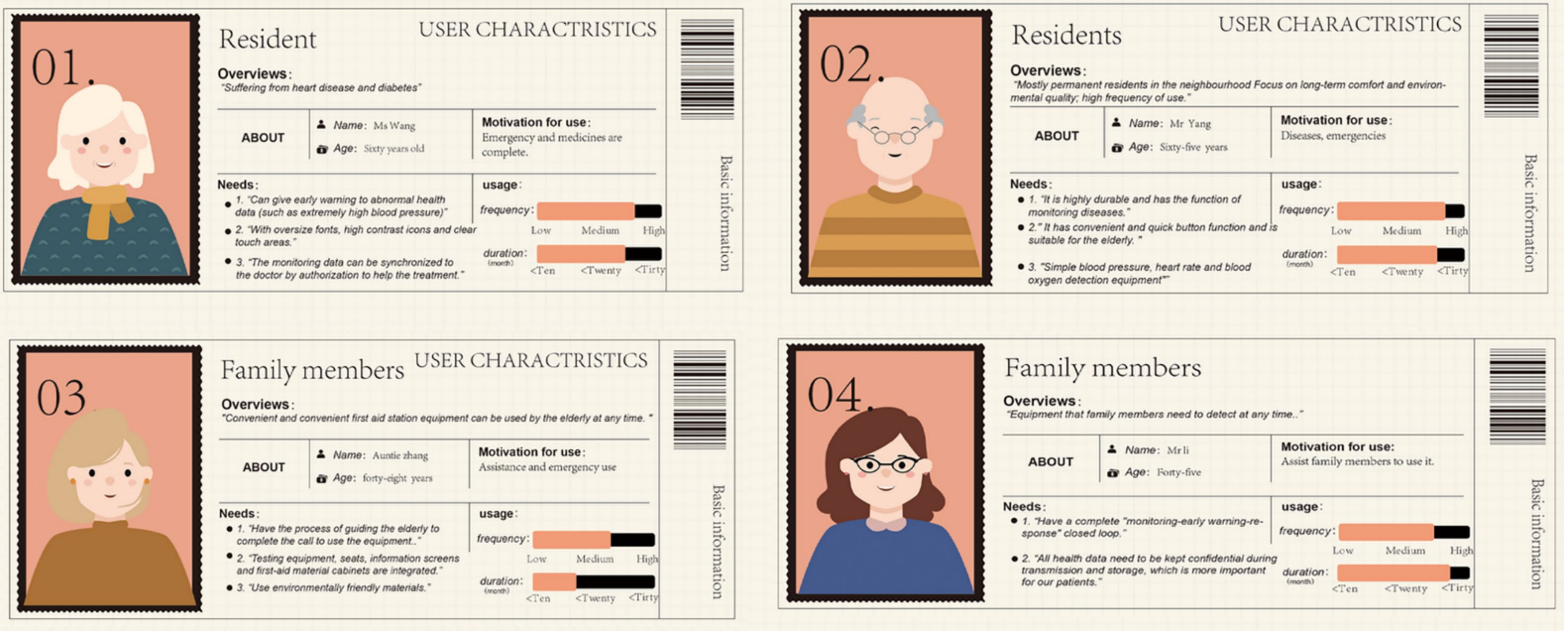
User images.

In order to improve the transparency of sample selection, the inclusion and exclusion criteria of the respondents are defined in Table 1. The inclusion criteria for the older adults samples are: they are over 60 years old, have lived in the above-mentioned communities for 6 months or more during the investigation period, have basic communication and understanding skills, and have informed consent to participate in the investigation. The inclusion criteria of family members or caregivers are: at least 18 years old, living together with the older adults, or taking on stable care responsibilities in daily life care, accompanying medical treatment, health management, etc. Exclusion criteria include: non-community permanent residents, those who cannot complete effective communication, those who are not fit to participate in the survey on that day, and those who have serious lack of questionnaire information or obviously abnormal answers. The portrait of the user is shown in Figure 2.

**Table 1:** Questionnaire on the basic situation of older adults residential buildings.

Category		Quantity	Proportion
Gender	Women	207	59%
	Male	144	41%
Age	30–60	66	18.8%
	60–70	197	56.1%
	70+	88	25.1%
Type of disease	Heart disease	139	39.6%
	Hypertension	101	28.8%
	Pneumonia	79	22.5%
	Osteoarthritis	32	1%

The research adopted a mixed-methods approach, combining structured questionnaires and semi-structured interviews to collect quantitative and qualitative data. The questionnaire was primarily used to identify the explicit needs of older adults residents and caregivers regarding community emergency facilities, covering dimensions such as basic information, health status, emergency knowledge, service expectations, and usage barriers, as shown in Table 1. The sample was recruited using a combination of purposive and convenience sampling, mainly conducted in community public activity spaces, health service-related venues, and residents’ daily activity areas, taking into account both weekdays and weekends. Considering the routines of older adults individuals, the survey was primarily scheduled for two time periods each day, 9:00–11:00 and 15:00–17:00, to enhance sample coverage and survey cooperation.

Based on the questionnaire survey, the study further employed semi-structured interviews to additionally explore usage barriers, situational needs, and potential preferences that questionnaires could not fully capture. The interviewees were selected using purposive sampling, covering both older adults residents and caregivers while ensuring diversity in age, health status, years of residence, and caregiving experience to enhance the diversity and representativeness of the data. The interview content primarily focused on topics such as experiences with community emergencies, difficulties in using existing facilities, ability to recognize and operate information, expectations for medical coordination, preferences for age-friendly interfaces, and community support needs.

**Figure 3:**
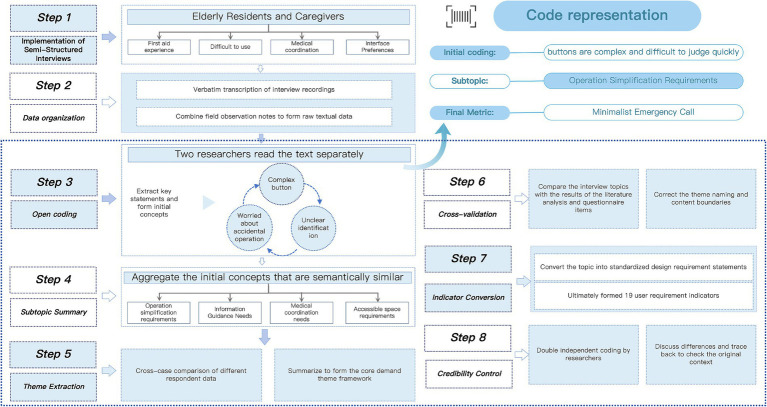
Interview data analysis flowchart.

During the data analysis process, the research team first transcribed all interview recordings verbatim and organized them together with field observation notes to form the raw textual data. Thematic analysis was then used to process the interview content in layers, as shown in the interview data analysis flowchart in Figure 3. First, two researchers independently read the text repeatedly, identified key statements related to the use of community emergency facilities based on the research objectives, and conducted open coding to extract initial concepts, such as “buttons are too complex to understand,” “afraid of making operation errors,” “want one-button contact with family or doctor,” and “cannot find equipment due to unclear labels.” Second, initial concepts with similar semantics or situational similarity were further aggregated into several sub-themes, such as “need for simplified operation,” “risk perception and concern about incorrect operation,” “need for information guidance,” and “medical coordination needs.” Finally, based on cross-case comparisons, the sub-themes were generalized and integrated, and cross-verified with literature analysis results and questionnaire items, ultimately identifying 19 user needs indicators, which served as the foundation for subsequent Kano classification, AHP weighting, and TRIZ design transformation.

To improve the credibility of the data analysis, two researchers independently carried out the initial coding and theme summarization, resolving coding discrepancies through discussion and negotiation; if necessary, the original interview context was revisited to minimize bias from the researchers’ subjective interpretations. Furthermore, after forming the final need indicators, the research team revised theme names and indicator descriptions by integrating literature and expert opinions to ensure they were more consistent with the analytical logic and expression standards of age-friendly community emergency facility design research.

**Figure 4:**
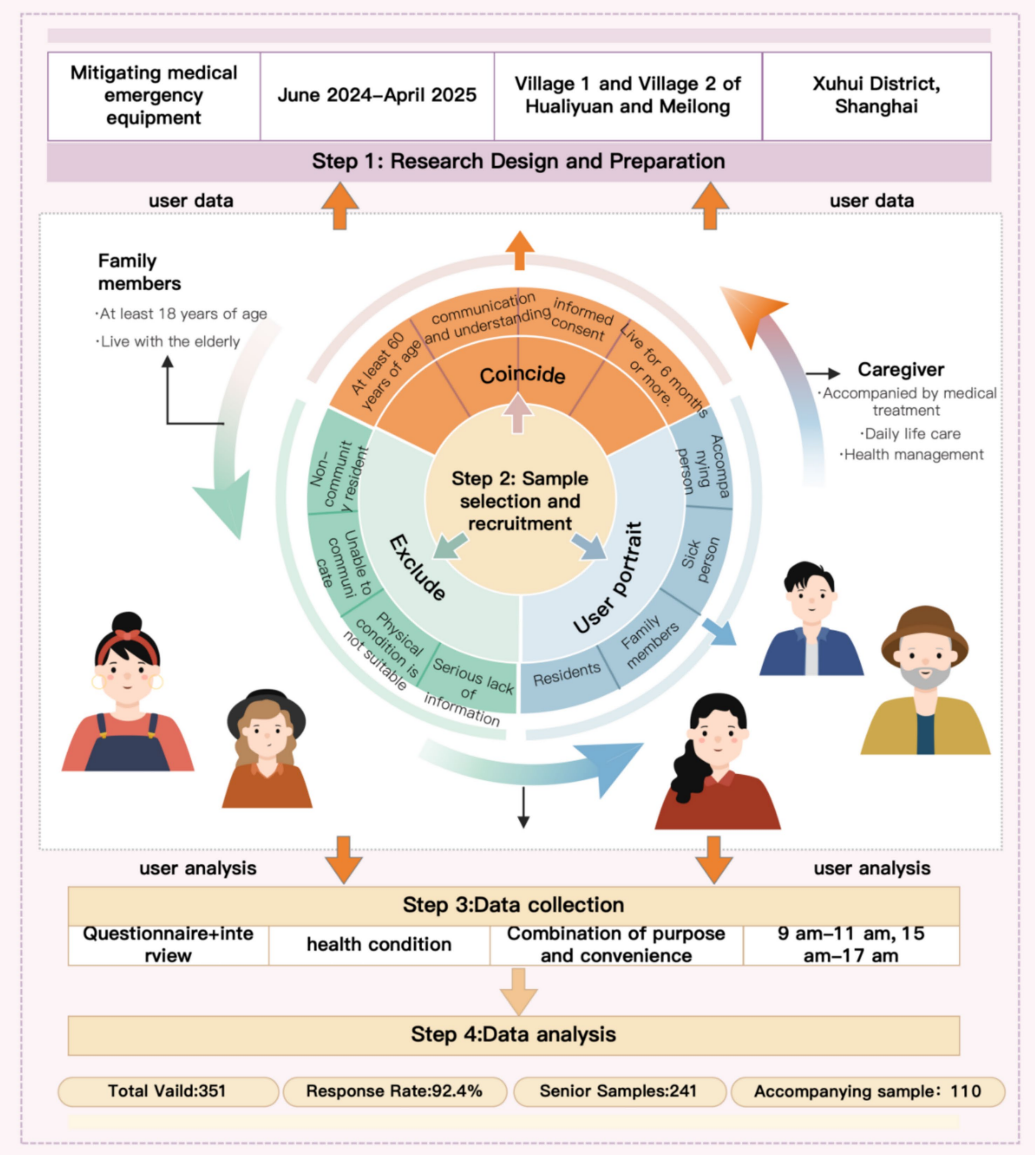
Survey user flow chart.

A total of 380 questionnaires were distributed in this survey, and 351 valid questionnaires were recovered, with an effective recovery rate of 92.4%. Among them, there are 241 samples from the older adults and 110 samples from family members or caregivers. The user survey flowchart is shown in Figure 4.

## 3 Method

### 3.1 KANO model analysis of age-friendly community emergency station user demand weight

Through interviews, user conversations, literature review, and expert consultations, it was found that the proportion of older adults people with diseases in this community is relatively high (42). Therefore, the emergency station will be equipped with AEDs, CPR masks/resuscitation masks, gauze, bandages, and other emergency supplies. Based on this, the user requirement indicators for the design of older adults-friendly health community emergency station products are classified and summarized in a hierarchical manner, forming requirement dimension items divided into four major categories (43, 44): A, B, C, and D, namely functional requirements, appearance requirements, cultural and management requirements, and technical requirements. The user requirement information in the requirement indicators is professionally expressed and classified to transform into standardized requirement element indicators. Among these, the requirement element indicators subdivided under these four categories cover a total of 19 specific items, with the specific evaluation indicators shown in Table 2.

**Table 2:** Evaluation indicators.

Demand latitude		Demand factor	Description
Functional requirements	A1	Minimalist emergency call	Entity big button
	A2	Basic vital signs monitoring	Simple blood pressure, heart rate and blood oxygen detection equipment
	A3	Clear guidance system	Guide the older adults to complete the whole process from calling to using the equipment.
	A4	Barrier-free and safe space	There is room for wheelchairs to turn around and avoid sharp corners.
	A5	Identity recognition and intelligent early warning	Brush the old age card, medical insurance card and other ways to quickly identify the user’s identity and automatically retrieve the basic medical history. The system can warn abnormal health data (such as extreme hypertension) and notify the preset emergency contact.
Appearance requirement	B1	Eye-catching visual design	Large illuminated sign
	B2	Safe and comfortable materials	Using eco-friendly materials that are gentle, non-slip, and antibacterial
	B3	Integrated functional layout	Integrate detection equipment, seats, information screens, and emergency supply cabinets into a unified system
	B4	Elder-friendly user interface	Featuring a minimalist design with oversized fonts, high-contrast icons, and clearly defined touch areas.
Culture and management needs	C1	Personalized health services	Users or their family members can customize health focus areas on the platform (such as focusing on blood pressure or blood sugar).
	C2	Community health platform integration	The community family doctor system and regional health cloud platform are connected, allowing monitoring data to be synchronized with doctors with authorization.
	C3	Environmental adaptation design	Good insulation, ventilation, and drainage performance
	C4	Easy to maintain and manage	Modular design
	C5	Promote community sharing and belonging	Incorporate elements of community culture and set up a community health bulletin board.
Technical requirements	D1	System intelligent integration	Form a complete ‘monitoring-warning-response’ closed loop
	D2	Reliability and stability	Equipped with remote fault diagnosis capabilities
	D3	Information visualization and minimalist operations	Strictly adhere to the principle of ‘one interface, one main task’ and reserve software and hardware interfaces for future new features (such as integrating new smart health devices and government service portals).
	D4	Modularity and scalability	Strictly adhere to the principle of ‘one interface, one main task’ and reserve software and hardware interfaces for future new features (such as integrating new smart health devices and government service portals).
	D5	Strict data privacy and security	All health data must be encrypted during transmission and storage.

#### 3.1.1 Analysis of questionnaire validity and reliability

Using SPSS software to conduct reliability and validity tests on the questionnaire, in terms of validity, the KMO method was used to examine whether the evaluation results can truly reflect the satisfaction elements of sustainable healthy community first aid stations (45). All the item test values were greater than 0.9, indicating that the questionnaire has good validity. The results of the questionnaire validity test are shown in Table 3, obtaining a KMO value of 0.912, Bartlett’s test of sphericity approximated *χ*^2^ = 3004.213, d*f* = 151, *p* < 0.001, indicating that the sample data is suitable for further structural analysis.

**Table 3:** Validity tests.

KMO and Bartlett’s tests		
KMO value		0.912
Bartlett’s test for sphericity	Approximate Chi-square	3004.213
	d*f*	151
	*p*-value	0

In terms of reliability, the results of the reliability test are shown in Table 4, where reliability analysis was carried out using Cronbach’s alpha coefficient, which is a commonly used method to test the reliability of questionnaire survey results. The reliability test of this questionnaire survey yielded a Cronbach’s *α* coefficient of 0.910. Since Cronbach’s coefficient is greater than 0.9, the questionnaire has good reliability.

**Table 4:** Reliability test.

Number of items	Sample size	Cronbach *α* coefficient
28	351	0.910

#### 3.1.2 Statistics and classification of user demand types

Based on the information in the table, as shown in Table 5, several different types of user needs can be clearly identified. Must-have needs are: minimalist emergency call (A1), basic vital signs monitoring (A2), clear guidance system (A3), prominent visual design (B1), integrated functional layout (B3), community health platform integration (C2), and intelligent system linkage (D1). Meeting these needs can significantly enhance user satisfaction (46, 47), while their absence or non-fulfillment may lead to a noticeable decline in user satisfaction (48).

**Table 5:** Statistics and classification of user demand types.

User needs elements (numbered)	A (%)	O (%)	M (%)	I (%)	R (%)	Q (%)	Better-worse classification	Better (%)	Worse (%)
(A1)	11.45%	31.23%	54.32%	3.54%	2.78%	4.98%	M	64.89%	−51.45%
(A2)	11.34%	32.16%	37.31%	20.78%	9.34%	1.78%	M	23.74%	−43.78%
(A3)	14.12%	39.11%	45.36%	11.43%	2.51%	4.88%	M	62.93%	−9.34%
(A4)	7.23%	42.21%	6.43%	22.76%	13.46%	1.17%	0	56.23%	−9.15%
(A5)	45.27%	0.33%	7.61%	19.89%	2.57%	0.42%	A	68.53%	−46.23%
(B1)	15.70%	0.00%	0.00%	39.67%	31.40%	13.22%	M	34.23%	0.00%
(B2)	32.41%	36.71%	11.01%	39.43%	6.45%	7.35%	O	50.30%	−67.23%
(B3)	19.43%	24.79%	29.76%	12.67%	2.91%	19.34%	M	21.10%	−23.45%
(B4)	41.17%	4.42%	32.40%	21.37%	2.98%	7.79%	A	34.03%	−4.22%
(C1)	56.12%	9.45%	4.13%	41.09%	4.32%	2.78%	A	45.34%	−10.35%
(C2)	21.72%	23.34%	31.64%	26.86%	8.99%	6.44%	M	26.34%	−34.12%
(C3)	1.59%	3.11%	7.53%	56.58%	21.59%	8.13%	I	24.21%	−2.00%
(C4)	26.45%	15.01%	20.83%	24.79%	5.79%	5.15%	A	12.24%	−45.75%
(C5)	17.88%	20.66%	19.43%	17.32%	6.74%	9.20%	O	34.87%	−45.78%
(D1)	47.35%	9.11%	12.83%	10.18%	12.79%	4.13%	M	11.67%	−34.45%
(D2)	1.65%	2.96%	7.92%	68.58%	21.22%	3.21%	I	46.23%	−4.01%
(D3)	41.17%	4.42%	32.40%	21.37%	2.98%	7.79%	A	34.03%	−4.22%
(D4)	3.23%	5.45%	4.99%	70.58%	14.32%	4.21%	I	45.29%	−2.70%
(D5)	17.11%	39.17%	12.76%	27.94%	3.41%	1.54%	O	58.31%	−6.78%

Attractive needs are: identity recognition and intelligent alerts (A5), age-friendly interactive interface (B4), personalized health services (C1), easy maintenance and management (C4), and information visualization with minimalist operation (D3). Meeting attractive needs can bring users unexpected pleasure and effectively improve overall satisfaction (49), so they should be highly considered and implemented as much as possible during the design process. One-dimensional needs are: accessible and safe spaces (A4), safe and comfortable materials (B2), promoting community sharing and belonging (C5), and strict data privacy and security (D5). The degree to which expected needs are met is positively correlated with user satisfaction and plays a major role in enhancing product attractiveness and improving user evaluations; hence, these needs should be prioritized and comprehensively fulfilled. Indifferent needs are: environment-adaptive design (C3), reliability and stability (D2), and modularity and scalability (D4). The presence or absence of these needs has no significant effect on user satisfaction, so they do not require excessive consideration (50, 51).

### 3.2 Demand weight analysis of older adults-friendly community first aid stations in AHP

Although the Kano model categorizes user needs for emergency station cabinet products in older adults-friendly communities, it still has limitations in presenting the weighting of the importance of various demand elements (52). In contrast, the Analytic Hierarchy Process (AHP), as a scientific and systematic tool for calculating weights, can effectively quantify the relative weights of user needs thereby improving the accuracy of demand analysis and the rationality of design decisions (53). Therefore, this paper introduces the AHP method to construct a clear hierarchy of user needs, taking key basic needs, performance needs, and attractive needs as the core levels of the evaluation system, and using the specific demand elements under them as sub-level content, ultimately forming a complete structure including the goal layer, criterion layer, and indicator layer, in order to comprehensively reflect the relative importance of various user needs in product design.

Based on the above user requirement hierarchy analysis diagram for calculation, the results of the weights of each matrix are shown in Tables 6–9. Consistency tests were also conducted, and the test results meet consistency requirements. The results of the consistency test are shown in Table 10.

**Table 10:** Matrix of normative layers.

Y	Essential attributes M	One-dimensional attributes O	Attractive attributes A	Weight value
Essential attributes M	1	1/2	1	0.3854
One-dimensional attributes O	1	1	2	0.2976
Attractive attributes A	1	1/2	1	0.1275

**Table 6:** Required requirements judgment matrix.

Y_M_	M1	M2	M3	M4	M5	M6	M7	Weight value
M1	1	1/2	1	1/2	1	1	1/2	0.1560
M2	2	1	1/2	1	1/3	1/2	1	0.1428
M3	1	2	1	1	1	1	1/2	0.1426
M4	2	1	2	2	1/2	2	1	0.0964
M5	1	3	1	2	1	1	1	0.0948
M6	1	2	1	1	1	1	1/2	0.1189
M7	2	1	2	1	1	1	1	0.1220

**Table 7:** Expected demand judgment matrix.

Y_O_	O1	O2	O3	O4	O5	Weight value
O1	1	1/3	1/2	1	1/2	0.2497
O2	3	1	1	1/3	1	0.1221
O3	2	1	1	1	1/2	0.2449
O4	1	1	1	1	1	0.1836
O5	2	2	2	1	1	0.2000

**Table 8:** Glamor demand judgment matrix.

Y_N_	N1	N2	N3	N4	Weight value
N1	1	1/2	1/3	1	0.2879
N2	2	1	1/2	1/4	0.2485
N3	3	2	1	1/2	0.1250
N4	1	4	2	1	0.1150

**Table 9:** Consistency test results.

Serial Number	$Y_{M}$	$Y_{O}$	$Y_{N}$
$\lambda_{\mathrm{MAX}}$	8.552	5.425	5.441
CI	0.079	0.106	0.110
CR	0.056	0.095	0.098

Based on the above calculation steps, data processing was carried out using SPSS statistical software, and the weights of each element in the criterion layer and its corresponding indicator layer in the evaluation index system were ultimately determined. Detailed results of the overall ranking were obtained. The comprehensive weight table of evaluation indicators is shown in Table 11, which indicates: the importance ranking of the indicators in the indicator layer is M > N > O, and the importance ranking of the indicators in the sub-criterion layer is M1 > O2 > M2 > O1 > O3 > M3 > N2 > O5 > N3 > O4 > N1 > M4 > M5 > N4. This indicates that users urgently need functions such as the establishment of age-friendly community emergency stations, intelligence, and reasonable activity space, providing a systematic solution for the effective use of older adults-friendly community emergency stations (54, 55).

**Table 11:** Table of combined weights of evaluation indicators.

Criterion layer	Sub-criteria level indicators	Sub-criterion layer weights	Comprehensive weight of indicators	Weighted sorting
M (1.410)	M1	0.1560	0.410	1
	M2	0.1428	0.310	3
	M3	0.1426	0.297	6
	M4	0.0964	0.021	12
	M5	0.0948	0.140	13
O (1.120)	O1	0.2497	0.364	4
	O2	0.1221	0.012	2
	O3	0.2449	0.035	5
	O4	0.1836	0.053	10
	O5	0.2000	0.102	8
N (2.216)	N1	0.2879	0.043	11
	N2	0.2485	0.259	7
	N3	0.1250	0.089	9
	N4	0.1150	0.004	14
	N5	0.2250	0.129	15

### 3.3 TRIZ demand weighting analysis of first aid stations in older adults-friendly communities

Design planning is carried out based on the demand weight ranking characteristics obtained previously, using TRIZ to resolve conflicts that arise during the design process (56). The contradiction matrix, composed of inventive principles and 39 technical parameters, is used to address technical and physical contradictions in the process of setting up emergency stations in older adults-friendly communities. Through analysis, four sets of conflicts were identified and transformed into TRIZ problem models, as shown in Table 12.

**Table 12:** Contradiction and conflict analysis and corresponding invention principles.

Contradictions and conflicts	Conflict types		Improve parameters	Deterioration parameters	Corresponding invention principle
Emergency power supply	Physical conflict	A1 (simplified emergency call system)	Power reliability	Equipment energy consumption/energy storage pressure	3.17.31.40
		D2 (reliability)			
Information sharing and privacy protection	Physical conflict	A5 (identity verification and early warning)	Information integrity	Information leak	19.31.32
		D5 (Data Privacy)			
Functional integration and spatial accessibility	Technical conflict	B3 (integrated layout)	34. Practicality (age-friendly)	36. System complexity (the contradiction between size and function)	36, 26, 12, 17
		A4 (accessible space)			
Intelligent automation and user control	Technical conflict	B4 (age-friendly interface)	43. Automation level	37. System security (due to misoperation or misunderstanding)	25.13.28.2.10.7
		C1 (personalized services)			

### 3.4 Strategy

#### 3.4.1 Safety and reliability strategy: constructing a physically safe environment that meets medical grade standards

In terms of safety and protection, this design has constructed a multi-layered defense system focused on the stable operation of medical equipment. The emergency station uses a redundant power supply architecture, integrating the main power system with a backup battery pack, ensuring that in the event of a sudden power outage, critical medical devices such as AEDs and vital signs monitors continue to receive power, guaranteeing that emergency procedures are not interrupted. All circuit systems are equipped with dual overload and surge protection to maintain the precision and reliability of medical-grade equipment. The cabin is made of fire-retardant materials with an IP54 sealed structure, and is equipped with an automatic temperature and humidity control system to provide a stable storage and working environment for internal consumables such as medications and blood glucose test strips, as well as precision instruments. All contact surfaces use antibacterial and easy-to-clean materials, with rounded edges for collision protection. The floor is covered with antibacterial and anti-slip mats, effectively preventing older adults users from slipping, bumping, or cross-infecting during emergency use, thereby creating a safe and reliable medical operation environment (57).

#### 3.4.2 Intelligent adaptability strategy: achieving a “monitoring-warning-intervention” medical closed-loop management

The system focuses on building a continuous health management service, integrating intelligent sensing and multimodal interaction to form a complete medical support chain from emergency response to daily management (58, 59). The core configuration includes an intelligent sensing system with fall detection capabilities, which can automatically initiate pre-hospital emergency procedures when a sudden fall of an older adults person is detected, simultaneously unlock emergency equipment such as AEDs and CPR masks, and guide rescue operations through voice and visual instructions to seize the “golden rescue window.” The interactive interface strictly follows human-machine ergonomics principles for medical devices, providing voice guidance, large fonts, high-contrast colors, and dialect recognition functions to ensure low cognitive load operations in emergency situations (60). The health monitoring module integrates medical-grade blood pressure monitors, oximeters, and glucose meters, with data uploaded in real time to the community health management platform, forming dynamic electronic health records. The system has intelligent interpretation and warning capabilities, automatically alerting risks and guiding the use of emergency supplies such as medical glucose tablets when detecting decreased blood oxygen levels or abnormal blood sugar. In addition, the intelligent medicine cabinet supports medication reminders and dosage dispensing, and links with the community pharmacy system to ensure continuous supply of chronic disease medications and adherence management, forming a complete service loop from acute event handling to long-term health management.

#### 3.4.3 Humanistic care design strategy: integrating older adults comprehensive assessment and psychological support care system

On the humanistic level, the design integrates the concept of comprehensive geriatric assessment, organically combining medical support with psychosocial services. The emergency station is equipped with a rest and observation area featuring older adults-friendly seats and assistive handrails, available for recovery after emergencies or routine health monitoring. The space uses clear color zoning and directional signage to help older adults individuals with mild cognitive impairment quickly identify functional areas (61, 62). A special “Peace of Mind Corner” service module is set up, providing emergency contact card generation, anti-wandering positioning devices, and direct access to psychological counseling hotlines, ensuring dual attention to the safety and mental state of the older adults. Community health screens dynamically update information on specialist doctor consultations, disease management courses, and rehabilitation activity schedules, guiding the older adults to actively participate in health management and enhancing their ability for self-care and social engagement (63).

#### 3.4.4 Community integration strategy: constructing a community health node within the tiered diagnosis and treatment system

At the level of community integration, emergency stations serve as the frontline nodes of the community-tiered medical care system, establishing a two-way referral and data-sharing mechanism with community health service centers, family doctors, and regional hospitals, achieving interconnected health records, coordinated follow-up management, and emergency response collaboration (64, 65). The stations set up volunteer service interfaces to support trained younger older adults volunteers in participating in daily operations and health education, building an intergenerational health mutual aid model within the community. By regularly conducting emergency skill training, chronic disease management classes, and specialized doctors’ community consultations, the emergency stations are developed into important bases for community health promotion and education, overall enhancing residents’ health literacy and the accessibility of primary medical services.

## 4 TOPSIS assessment

To improve the relevance and interpretability of the plan evaluation, this study uses the TOPSIS method (66–70) to make a comprehensive decision on the design plans of older adults-friendly health community first-aid stations. In this method, 15 demand factors are used as evaluation indicators for the plans, and all are set as positive indicators. This evaluation stage employs a dual-subject assessment method combining an expert group and a user group. The expert group is mainly used to judge the comprehensive performance of the design plans from a professional perspective in terms of older adults-friendliness, emergency response, information linkage, interface usability, and implementation feasibility. The user group mainly reflects the actual perception and subjective preferences of the target user population, focusing particularly on interface recognition, ease of operation, spatial adaptability, and overall acceptability, which are closely related to the user experience. By incorporating both types of evaluation subjects, it is possible to mitigate the bias that may arise from a single evaluation perspective, ensuring that the evaluation results consider both professionalism and user experience.

### 4.1 Sample selection

The expert group was selected using purposive sampling. Inclusion criteria for participants required relevant professional backgrounds in industrial design, age-friendly design, community health services, emergency medicine, or public service management, with at least 5 years of related research, practice, or management experience, and familiarity with the needs and characteristics of the older adults population as well as the evaluation of community health service facilities. The user group was recruited from the target user population. Inclusion criteria included being aged 60 or above, having experience living in the community, possessing basic cognitive comprehension and expression abilities, being able to independently complete evaluation scales, and giving informed consent to participate in the study.

Regarding the sample size, this study included a total of 20 experts and 20 older adults users. It should be noted that the main purpose of the TOPSIS evaluation phase was not to conduct large-sample statistical inference but rather to perform a multi-criteria comparative analysis of design prototypes and evaluation samples based on an established indicator system. Therefore, the emphasis was placed more on the relevance, experience, and comparability of the evaluators than on the representativeness of sample size. The inclusion of 20 experts and 20 older adults users allows, on the one hand, the consideration of both professional judgment and target user experience within the feasible research implementation conditions, and on the other hand, helps achieve relatively stable scoring results during the prototype evaluation phase, thereby enhancing the reliability and interpretability of scheme comparison.

Furthermore, considering that the prototypes proposed in this study involve multiple dimensions, including emergency facility function configuration, older adults-friendly interaction design, and community health service linkage, relying solely on expert evaluation may overlook the perceptual differences of older adults users in real-use scenarios, while relying solely on user evaluation may make it difficult to fully assess the professional and implementation rationality of the schemes. Therefore, a dual-group evaluation structure with 20 experts and 20 older adults users helps more comprehensively reflect the comprehensive performance of design plans in terms of both “professional feasibility” and “user acceptability.” The results of this phase are mainly used to compare the relative merits of different schemes under the evaluation framework of this study, and the conclusions should be understood as evidence of small-scale assessment sample preferences for prototype schemes rather than being directly generalized to the broader community population.

**Figure 5:**
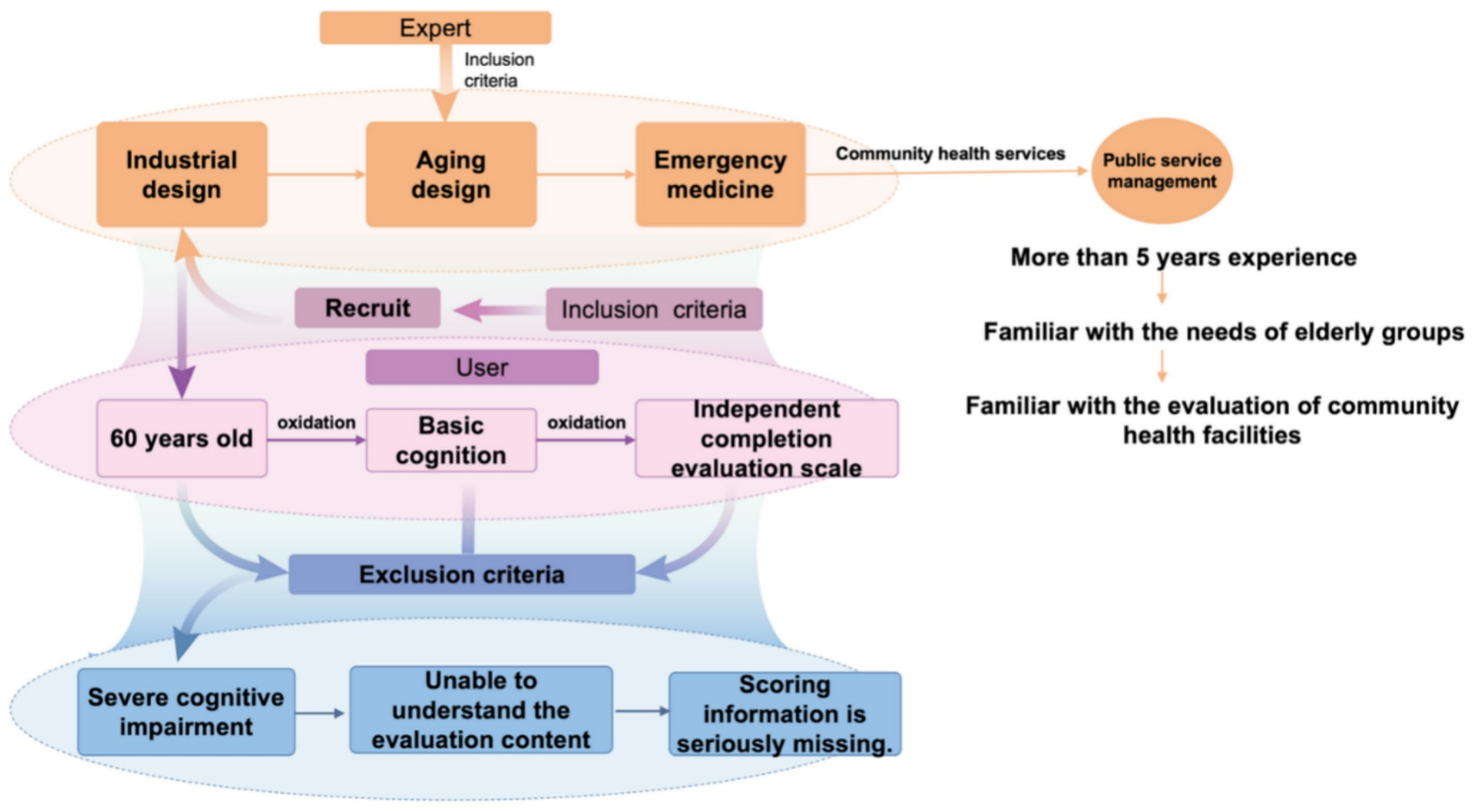
User selects analysis chart.

**Figure 6:**
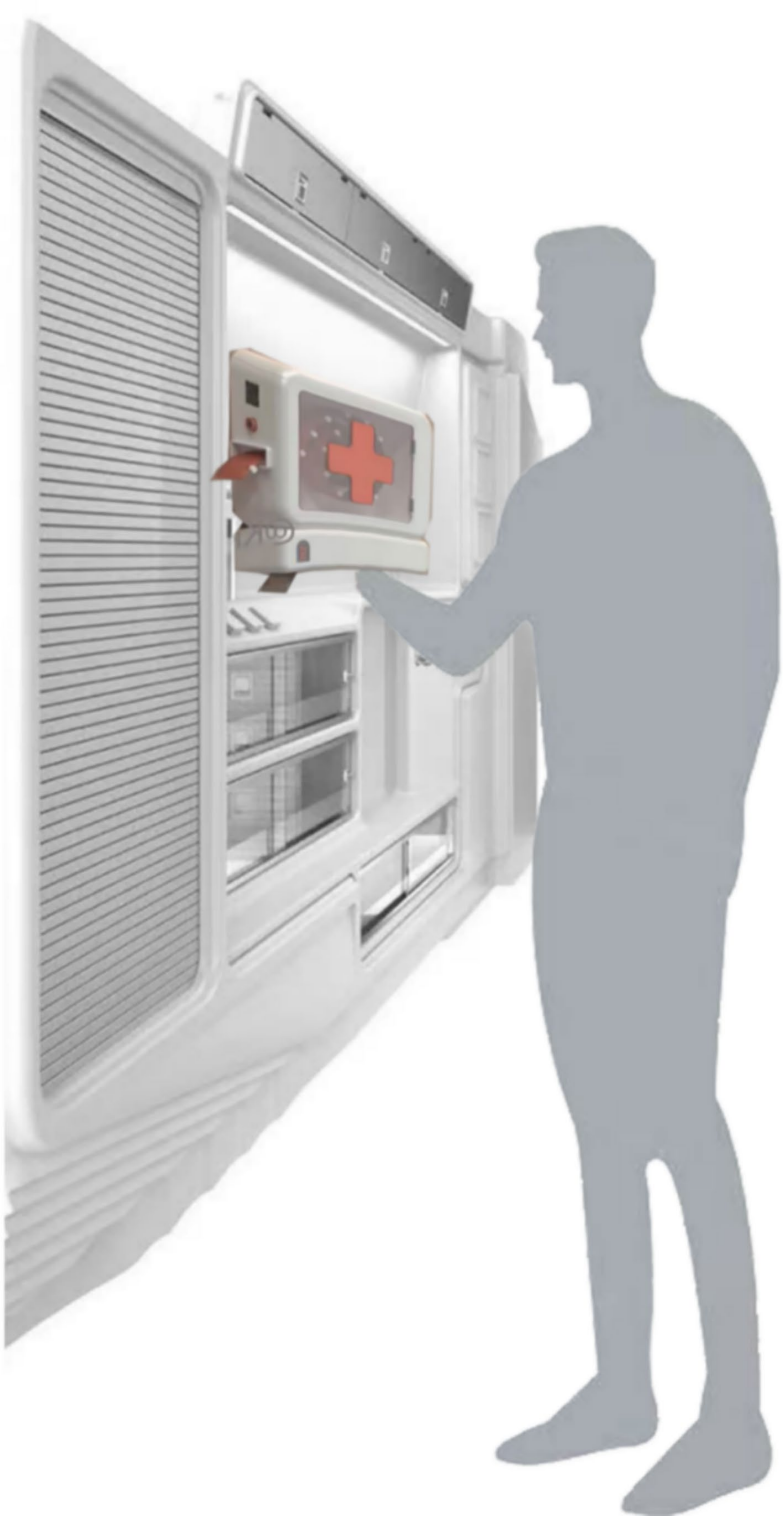
Family user interaction diagram.

During the evaluation process, experts and users rated the design schemes and the evaluation sample schemes shown in Figure 5 based on 15 evaluation indicators. A seven-point Likert scale was used, where scores from 1 to 7 represented “very poor,” “poor,” “not good,” “average,” “good,” “very good,” and “excellent,” respectively. After obtaining all scoring results, the evaluation data from the expert and user groups were aggregated, and the average score for each indicator was calculated to form the initial evaluation matrix *F*, with specific results shown in Table 13. This matrix provides the foundational data support for the subsequent TOPSIS comprehensive evaluation and scheme ranking (see Figure 6).

**Table 13:** Initial evaluation matrix.

F_M1_	F_M2_	F_M3_	F_M4_	F_M5_	F_O1_	F_O2_	F_O3_	F_O4_	F_O5_	F_N1_	F_N2_	F_N3_	F_N4_	F_N5_
5.6	3.6	5.1	3.2	4.5	4.7	3.9	5.1	5.6	4.9	3.7	4.6	5.2	4.8	4.3
5.8	2.9	4.6	4.7	5.1	2.7	4.5	4.1	3.8	3.6	4.6	4.2	3.8	5.1	4.7

### 4.2 Sample analysis

Based on the above, after the expert group and the older adults user group completed scoring on various evaluation indicators, this study further employed the TOPSIS method for multi-criteria comprehensive analysis of the prototype schemes and evaluation samples. Specifically, the scoring results of the two groups of evaluators on each indicator were first summarized, and the average value of each indicator was calculated to form the initial evaluation matrix *F*. This matrix reflects the original performance of different schemes on each evaluation indicator and serves as the basis for subsequent comprehensive ranking. Subsequently, to eliminate the impact of differences in units and scoring scales of different indicators on the results, the initial evaluation matrix was standardized to obtain a dimensionless standardized matrix.

Based on the standardization, combined with the indicator weights obtained from the previous AHP analysis, the standardized matrix was weighted to construct a weighted standardized matrix, reflecting the relative importance of different evaluation indicators in scheme comparison. Then, according to the setup where all indicators are positive, the positive ideal solution and negative ideal solution for each scheme were determined. The positive ideal solution represents a hypothetical scheme where all indicators reach their optimal values, while the negative ideal solution represents a hypothetical scheme where all indicators are at their worst values.

According to the following TOPSIS method, by calculating the positive and negative ideal solutions and the relative closeness of each scheme, the specific implementation steps are as follows:

(1) Perform averaging treatment to convert the questionnaire results into the initial evaluation matrix.
(2) Normalize the initial evaluation matrix to obtain the standardized matrix $R_{\mathrm{ij}}$ .

$$R_{\mathrm{ij}}=\frac{f_{\mathrm{ij}}}{\sum_{i=1}^{m}f_{\mathrm{ij}}^{2}}\;\;i=1,2,\ldots ,m;j=1,2,\ldots ,n$$

(3) Using the target weights of various evaluation indicators, calculate the weighted normalized matrix $u_{\mathrm{ij}}$ .

$$u_{\mathrm{ij}}=W_{j}R_{\mathrm{ij}}$$

in the formula: $W_{j}$ It is the weight.

(4) Calculate the positive ideal solution $A^{+}$ and the negative ideal solution $A^{-}$ .

$$M_{j}^{+}=\max\{u_{1j},u_{2j},\ldots ,u_{\mathrm{nj}}\}\;\;j=1,2,\ldots ,m$$

$$M_{j}^{-}=\min\{u_{1j},u_{2j},\ldots ,u_{\mathrm{nj}}\}\;\;j=1,2,\ldots ,m$$

(5) Calculate the result according to the above formula.

$$A^{*}=(M_{1}^{+},M_{2}^{+},\ldots ,M_{m}^{+})$$

$$\bar{A}=(M_{1}^{-},M_{2}^{-},\ldots ,M_{m}^{-})$$

(6) Calculate the distance of each scheme to the ideal solution using Euclidean distance, where $S_{i}^{+}$ represents the distance to the positive ideal solution, and $S_{i}^{-}$ represents the distance to the negative ideal solution.

$$s_{i}^{+}=\sqrt{\sum_{j=1}^{n}{\left(u_{\mathrm{ij}}-u_{j}^{+}\right)}^{2}}\;\;i=1,2,\ldots ,m$$

$$S_{i}^{-}=\sqrt{\sum_{j=1}^{n}{\left(u_{\mathrm{ij}}-u_{j}^{-}\right)}^{2}}\;\;i=1,2,\ldots ,m$$

(7) Calculate the relative closeness $C_{i}$ of each scheme to the ideal solution

$$C_{i}=\frac{S_{i}^{-}}{i=1,2,3\ldots ,m}\;\;i=1,2,3,\ldots ,m$$

On this basis, the Euclidean distances between each evaluation plan and the positive ideal solution and negative ideal solution are calculated respectively, to measure the extent to which each plan is close to the optimal state and far from the worst state. Finally, based on the distances to the positive and negative ideal solutions, the relative closeness $C_{i}$ of each plan is calculated. The larger the $C_{i}$ value, the closer the plan is to the positive ideal solution and the farther from the negative ideal solution, indicating better overall performance. Conversely, a smaller $C_{i}$ value indicates that the plan’s overall performance is relatively weaker.

According to the calculation results of this study, the relative closeness of the design plan is 0.894, while that of the evaluation sample is 0.167, indicating that under the evaluation indicator system established in this study, the design plan is closer to the ideal state compared to the evaluation sample, and is therefore considered to have superior overall performance. It should be noted that this result mainly reflects the relative preference and comprehensive judgment of a small-scale evaluation sample for different plans, supporting the comparative ranking between prototype plans, and should not be directly extrapolated to actual application effects on a larger community scale. Table 14 clearly indicates that the CI value of the design plan is at the highest level, and therefore it is judged to be the best plan. In addition, the ranking of the evaluation samples is lower than that of the design plan based on Kano-AHP-TRIZ-TOPSIS, showing that the user-demand-based design path for older adults-friendly health community first aid station products proposed in this study demonstrates good potential in terms of feasibility.

**Table 14:** Positive and negative ideal solutions and relative closeness progress.

Evaluation object	Positive ideal solution	Negative ideal solution	Relative closeness	Sort
Design scheme	0.259	3.211	0.894	1
Evaluation sample	1.682	0.549	0.167	2

## 5 Discussion

### 5.1 Theoretical significance

The theoretical significance of this study is mainly reflected in the integration of methods and the construction of an analytical pathway for the design of emergency facilities in older adults-friendly communities. Addressing issues in traditional public facility design, such as rough identification of user needs, unclear prioritization of needs, and difficulty in systematically handling design conflicts, this study attempts to sequentially integrate the Kano model, the AHP method, TRIZ theory, and the TOPSIS method to form an analytical framework consisting of “needs identification—weight ranking—conflict transformation—design evaluation.” The results indicate that this framework can clearly support the hierarchical identification of older adults users’ needs, selection of key requirements, and transformation of design elements in the case studied, providing a logical research pathway for the prototyping of older adults-friendly community emergency stations.

At the same time, in the design of public facilities in highly older adults communities, relying solely on experiential judgment or a single method often makes it difficult to balance the complexity of needs with the feasibility of design. The integration of multiple methods helps improve the coherence of the analysis process and the transparency of design decisions. Especially when facing scenarios involving differences in older adults users’ operational capabilities, limited community space, and the coexistence of demands for linked health services, the integrated framework can help researchers understand problems from multiple dimensions and form more targeted design responses to some extent. The value of the methods discussed in this study is primarily reflected in their applicability and inspirational significance in this case. However, their stability and general applicability across different community types and facility scenarios still require further verification in subsequent research.

### 5.2 Practical significance

The practical significance of this study is mainly reflected in providing a user-oriented prototype design approach for the age-friendly optimization of emergency facilities in older adults communities. The research is based on field investigations of three old communities in Xuhui district, Shanghai, identifying and summarizing the key concerns of older adults residents and caregivers in aspects such as emergency response, operation recognition, medical coordination, spatial adaptation, and community support. The resulting prototype scheme is not a simple accumulation of individual functions but attempts to establish a relatively coordinated design relationship between safety and reliability, intelligent adaptability, humanistic care, and community integration.

From the perspective of design practice, this prototype has certain reference value in several aspects. First, through integrated layout, modular organization, and accessibility detail handling, it attempts to address the issues of limited public space and dispersed facility layout in older adults communities. Second, through large-font high-contrast interfaces, multimodal interaction, and simplified operation processes, it attempts to reduce the cognitive load for older adults users during recognition and usage. Third, by connecting with health platforms, providing information prompts, and embedding community sharing elements, it explores the potential interface between emergency facilities and community daily health services. The TOPSIS evaluation results indicate that the prototype achieved a relatively high overall preference under the evaluation framework set in this study, suggesting that it has certain advantages at the scheme comparison level. This advantage mainly reflects the evaluators’ relative preference for the prototype, so the practical significance of this study is better understood as a reference for prototype development, preliminary scheme selection, and older adults-friendly design optimization, rather than a direct demonstration of effectiveness in actual community application.

## 6 Limitation

Although this study has achieved positive results, there are still the following limitations, which point to directions for future research:

(1) Limitations of the research sample’s region and size: The empirical study was limited to three specific old residential communities in Xuhui District, Shanghai, with a limited sample size and geographic scope. The applicability of the research conclusions to older adults communities in different regions and cultural backgrounds remains to be further verified.
(2) Insufficient coverage of long-term operation and maintenance costs: The study focuses on the plan generation and evaluation during the design phase, while the discussion on sustainability issues such as long-term operation models after construction, cost sharing for maintenance, and technology iteration and upgrades is still not in-depth.
(3) Practical barriers to technology implementation: The proposed intelligent system integration (such as seamless connection with regional health cloud platforms) may face challenges in reality, including data interface standards, administrative barriers, and network security. These practical implementation difficulties were not fully discussed in the study.

## 7 Conclusion

This study focuses on the public health service needs of aging communities in highly aged urban areas, taking Huali Yuan, Meilong First Village, and Meilong Second Village in Xuhui District, Shanghai as empirical cases. It constructs and applies the “Kano-AHP-TRIZ-TOPSIS” integrated design framework to conduct a systematic study and prototype design of older adults-friendly community first-aid stations. Based on the results of needs analysis, design translation, and prototype evaluation, the main conclusions can be summarized in the following three levels:

### 7.1 Methodological aspect

Proposes a systematic approach for the design of older adults-friendly community emergency facilities.

This study shows that serially integrating the Kano model, AHP method, TRIZ theory, and TOPSIS evaluation can form a research pathway from user needs identification, prioritization of requirements to transformation of design conflicts. Compared with design processes dominated by a single method, this integrated framework helps enhance the hierarchical analysis of requirements and the systematicness of design decisions, providing clearer methodological support for the prototyping of complex community public health facilities. It should be noted that the “effectiveness” reflected in this study mainly refers to its applicability and operability in the current case study; further testing in more community scenarios is needed to verify its stability and generalizability.

### 7.2 Design aspect

Forming a prototype design approach oriented toward the needs of old communities.

Based on the results of demand analysis and conflict transformation, this study extracts four design focus areas: safety and reliability, intelligent adaptability, humanistic care, and community integration. On this basis, a prototype plan for an older adults-friendly community first-aid station was developed. This prototype attempts to address practical issues in old communities, such as limited space, differences in older adults users’ operational abilities, and insufficient linkage with community health services. For example, it balances functional configuration and accessibility needs through integrated layouts and modular design, enhances older adults users’ recognition and operational convenience through multimodal interactions and simplified interfaces, and strengthens the connection between the facility and everyday community service scenarios through health platform integration and community information embedding. The above results indicate that this prototype possesses a certain level of demand responsiveness and solution integration potential at the design level.

### 7.3 Application level

The prototype scheme shows certain potential for community integration and practical reference value.

Under the evaluation framework set in this study, the prototype scheme received higher overall preference compared to the evaluated samples, indicating that it has certain advantages in emergency response convenience, interaction usability, and medical linkage support. This suggests that older adults-friendly emergency facilities aimed at older communities can not only serve as emergency support nodes in sudden situations but also potentially play a more comprehensive service role within the community health service system. However, it should be emphasized that the evaluation results in this study are primarily based on comparative assessments from a small sample of experts and older adults users, reflecting relative preferences of different schemes under specific indicators, and cannot be directly equated with the actual usage effect after real community deployment. Broader impacts, such as improvement in health literacy, enhancement of social support, or strengthening of community medical resilience, still need to be verified through subsequent field applications and long-term follow-up.

In summary, this study provides a user demand-oriented approach for analyzing and transforming the design of emergency facilities suitable for older adults residents in older communities, and also offers a reference for the prototype development and program evaluation of community health service points. Its significance is more reflected in the integration of methods, design inspiration, and preliminary prototype comparison, while the actual effectiveness of its application in a broader range of communities still requires further research and verification.

## Data Availability

The raw data supporting the conclusions of this article will be made available by the authors, without undue reservation.
